# The crosstalk between subjective fibromyalgia, mental health symptoms and the use of over-the-counter analgesics in female Syrian refugees: a cross-sectional web-based study

**DOI:** 10.1007/s00296-023-05521-0

**Published:** 2024-01-29

**Authors:** Omar Gammoh, Alaa A. A. Aljabali, Murtaza M. Tambuwala

**Affiliations:** 1 Department of Clinical Pharmacy and Pharmacy Practice, Faculty of Pharmacy, Yarmouk University, PO BOX 566, Irbid, 21163 Jordan; 2 Department of Pharmaceutics and Pharmaceutical Technology, Faculty of Pharmacy, Yarmouk University, PO BOX 566, Irbid 21163, Jordan; 3 Lincoln Medical School, University of Lincoln, Brayford Pool Campus, Lincoln, LN6 7TS UK; 4 College of Pharmacy, Ras Al Khaimah Medical and Health Sciences University, Ras Al Khaimah, UAE

**Keywords:** Female, Fibromyalgia, Mental health, Refugees, Surveys and questionnaires, Syria

## Abstract

Suboptimal fibromyalgia management with over-the-counter analgesics leads to deteriorated outcomes for pain and mental health symptoms especially in low-income countries hosting refugees. To examine the association between the over-the-counter analgesics and the severity of fibromyalgia, depression, anxiety and PTSD symptoms in a cohort of Syrian refugees. This is a cross-sectional study. Fibromyalgia was assessed using the patient self-report survey for the assessment of fibromyalgia. Depression was measured using the Patient Health Questionnaire-9, insomnia severity was measured using the insomnia severity index (ISI-A), and PTSD was assessed using the Davidson trauma scale (DTS)-DSM-IV. Data were analyzed from 291. Among them, 221 (75.9%) reported using acetaminophen, 79 (27.1%) reported using non-steroidal anti-inflammatory drugs (NSAIDs), and 56 (19.2%) reported receiving a prescription for centrally acting medications (CAMs). Fibromyalgia screening was significantly associated with using NSAIDs (OR 3.03, 95% CI 1.58–5.80, p = 0.001). Severe depression was significantly associated with using NSAIDs (OR 2.07, 95% CI 2.18–3.81, p = 0.02) and CAMs (OR 2.74, 95% CI 1.30–5.76, p = 0.008). Severe insomnia was significantly associated with the use of CAMs (OR 3.90, 95% CI 2.04–5.61, p < 0.001). PTSD symptoms were associated with the use of CAMs (β = 8.99, p = 0.001) and NSAIDs (β = 10.39, p < 0.001). Improper analgesics are associated with poor fibromyalgia and mental health outcomes, prompt awareness efforts are required to address this challenge for the refugees and health care providers.

**Supplementary Information:**

The online version contains supplementary material available at 10.1007/s00296-023-05521-0.

## Introduction

Migration resulting from war displacement has become a growing global challenge, with the number of international refugees reaching unprecedented levels [1]. Among traumatized refugees, chronic pain is frequently reported, contributing to a detrimental cycle of poor mental health symptoms and low quality of life [2, 3], and is part of a vicious cycle of poor mental health symptoms and low quality of life [4].

Fibromyalgia, a condition characterized by widespread pain accompanied by mood and sleep disturbances, is prevalent in approximately 5% of the general population and affects around 30% of war-displaced Syrian refugees, with a higher incidence in women [5–7].

Fibromyalgia significantly impairs daily functioning and social activities in women and is closely associated with poor mental health status. A substantial proportion of fibromyalgia patients also experience symptoms of post-traumatic stress disorder (PTSD), with approximately one in every two patients reporting clinically significant PTSD and insomnia [4, 8]. Depression and anxiety disorders are also commonly observed, affecting 40% of individuals diagnosed with fibromyalgia [9].

The management of fibromyalgia is multifaceted and holistic, encompassing psychotherapy, physiotherapy, and pharmacotherapy [10]. The American College of Rheumatology (ACR) guidelines recommend the use of centrally acting medications (CAMs), including anti-seizure medications and serotonin-norepinephrine reuptake inhibitors, as they have shown efficacy in fibromyalgia treatment [11]. However, due to a lack of awareness about fibromyalgia, over-the-counter (OTC) analgesics such as acetaminophen (APAP) and non-steroidal anti-inflammatory drugs (NSAIDs) are commonly utilized [7]. In addition, individuals may resort to various household pain remedies, such as herbal preparations or warm compresses, to alleviate the widespread pain associated with fibromyalgia [12].

Proper diagnosis, management, and long-term care for women with fibromyalgia require specialized expertise, laboratory tests, medications, psychotherapy, social support, physiotherapy, and other interventions. Unfortunately, these resources are often unavailable or overlooked, particularly in war-displaced refugee communities where priority is given to addressing basic medical needs [13]. As a result, the majority of Syrian women tend to rely on conventional analgesics to manage their symptoms [12]. However, the potential impact of using these analgesics on fibromyalgia symptoms and related mental health outcomes remains largely unknown.

To the best of our knowledge, limited data exist regarding the potential association between analgesics such as APAP, NSAIDs, CAM, and the symptoms of fibromyalgia, depression, insomnia, and PTSD in vulnerable female refugees. Therefore, the objective of this study is to examine the association between OTC analgesics and the severity of subjective fibromyalgia, depression, insomnia, and PTSD symptoms in a cohort of war-displaced female Syrian refugees residing in Jordan.

## Methods

This is a cross-sectional cohort study using predefined inclusion criteria. It followed a similar design as in previous cross-sectional cohort studies [14–16].

### Patients

The study recruited female Syrian refugees through the utilization of data sets from Caritas, a Catholic Non-governmental organization, which operates medical and social care centers in Jordan.

### Inclusion criteria

Females, aged above 18 years old, frequenting Caritas Primary Social and Health Care Centers, living in urban districts, displaced for 5 years or more, non-smoking, unemployed, willing to participate, and have completed the study questionnaire were included in the study.

### Exclusion criteria

Females, aged below 18 years old, displaced in Jordan for less than 5 years, employed, smoking, unwilling to participate, and not completing the study questionnaire were excluded from the study.

### Main outcome variables

#### Fibromyalgia

To screen for fibromyalgia symptoms among the refugees, the Patient Self-Report Survey for Fibromyalgia Assessment was employed. Diagnosing fibromyalgia accurately is challenging, particularly in low-income settings in primary care centers serving a high number of refugees where the care is focused on controlling infectious diseases and non-communicable chronic diseases such as hypertension and diabetes [13, 17]. Therefore, the authors opted to use the aforementioned scale, which comprises the Widespread Pain Index (a body map depicting 19 potential tender points) and Symptom Severity. Based on the 2011 modification of the ACR preliminary diagnostic criteria for fibromyalgia, individuals who reported experiencing symptoms at a similar level for at least 3 months, with a score equal to or greater than 13 points (7 or higher points on the widespread pain scale and 5 or more on the severity scale and symptoms that persist for 3 months) and the absence of a medical condition explaining the symptoms, were deemed to have fibromyalgia [18]. This approach was further informed by previous research that examined the prevalence of fibromyalgia using questionnaire-based methods [14–16].

#### PTSD

To assess the severity of post-traumatic stress disorder (PTSD) among participants, the Davidson Trauma Scale (DTS) [19] based on DSM-IV criteria was employed. The Arabic version of the DTS, used by Ali et al. [20], was utilized. This 17-item self-report measure evaluates the 17 symptoms of PTSD outlined in the DSM-IV. Each item is rated on a 5-point frequency scale (ranging from 0 for "not at all" to 4 for "every day") and a severity scale (ranging from 0 for "not at all distressing" to 4 for "extremely distressing"). Scores on the DTS range from 0 to 68, with higher scores indicating more severe PTSD symptoms.

#### Depression

Depression severity was assessed using the Arabic-validated version of the Patient Health Questionnaire-9 (PHQ-9), which aligns with the Diagnostic and Statistical Manual of Mental Disorders-IV criteria for diagnosing depressive symptoms [21]. The PHQ-9 has demonstrated a sensitivity and specificity of 88% for severe depression. It measures depression symptoms over the past 2 weeks and yields a score between 0 and 27, with a cutoff score of > 14 indicating severe depression [22–24].

#### Insomnia

The Arabic version of the Insomnia Severity Index (ISI-A) was used to evaluate sleep quality. Developed by [25], the ISI consists of 7 Likert-type questions, and its score ranges from 0 to 28. A cutoff score of > 14 indicates severe insomnia symptoms. The ISI has been validated for use in the Arabic language [26]

### Study factors

#### Covariates

A self-administered structured online questionnaire was employed, consisting of various sections. Participants provided information on their age, marital status, residence location, body mass index, presence of chronic diseases namely hypertension and diabetes, and utilization of over-the-counter analgesics or centrally-acting prescribed medications for pain related to fibromyalgia symptoms.

#### Exposure

To ensure accurate data regarding analgesics used, the questionnaire incorporated brand and generic names, as well as pictures, of analgesics dispensed in Caritas pharmacies. This encompassed acetaminophen (APAP), non-steroidal anti-inflammatory drugs (NSAIDs), and centrally acting medications (CAMs) such as anti-seizure medications or serotonin-norepinephrine reuptake inhibitors (e.g., duloxetine), depending on availability in the Caritas pharmacy unit. Additionally, two additional options were included: hot water compresses and herbal homemade remedies like anise, fennel, and green tea used in households.

### Procedures

A well-trained female research assistant contacted potential participants via phone calls to provide detailed explanations regarding the study's objectives and methods. Interested individuals were then sent a study link that directed the participants to the Google survey to participate in the study.

The first page of the study instrument consisted of the informed consent form, where participants were required to read the objective and the methods of the study and then choose either to (“Yes: I agree to participate” or “No: I don’t agree to participate”). The study instrument was prepared in Arabic language. It is important to note that participants had the right to withdraw from the study at any time. The study received approval from the Yarmouk University IRB committee (number 175/2023) dated in 30/5/2023].

### Statistical analysis

Descriptive statistics, including frequencies, means, and standard deviations, were utilized to summarize sample characteristics. Multivariate binary logistic regression models were employed to examine the association between different analgesics and mental health outcomes, such as depression, anxiety, and insomnia. For PTSD, a multivariate linear regression model was employed to assess PTSD severity, preceded by an independent t test to examine the association between analgesic groups and PTSD severity, as the scale used did not establish a cutoff score. Categorical variables were analyzed using chi-square analysis, and variables with a p value < 0.10 were included in the multivariate regression models. Finally, multivariable regression models were performed to identify independent associations between exposure variables and outcome variables (dependent variables). A statistical significance level of p < 0.05 (two-sided) was set, and estimates were provided with a 95% confidence interval.

## Results

### Response rate

Out of the 1100 eligible participants approached over the phone, 543 phone numbers were disconnected, 219 declined participations, and 47 were excluded due to incomplete data. Therefore, a total of 291 participants were included for analysis, resulting in a response rate of 26.5%.

### Participants characteristics

Among the 291 participants, 151 (51.9%) were aged above 35 years old, 232 (79.7%) were married, and 169 (58.1%) resided outside Amman. Regarding analgesic use, 221 (75.9%) reported using acetaminophen, 106 (36.4%) reported using household herbal remedies, 79 (27.1%) reported using non-steroidal anti-inflammatory drugs (NSAIDs), 42 (14.4%) reported using hot compresses, 56 (19.2%) reported receiving a prescription of centrally acting medications (CAMs), and 24 (8.2%) reported using no analgesics. Moreover, 169 (58.1%) reported a score consistent with fibromyalgia symptoms, 170 (58.4%) reported a score consistent with severe depressive symptoms, 159 (54.6%) reported a score consistent with severe insomnia symptoms, and the mean score for post-traumatic stress disorder (PTSD) was 30.57 (± 18.06). Please refer to Table 1 for detailed information.

**Table 1:** Demographics and clinical data of the participants

Factor	Category	n (%)
Age	Below 35 years	140 (48.1%)
	35 and above	151 (51.9%)
Marital status	Single	59 (20.3%)
	Married	232 (79.7%)
Education	Below high school	163 (56%)
	High school and above	128 (44%)
Residence	Amman	122 (41.9%)
	Peripheries	169 (58.1%)
Body mass index	25 and below	81 (27.8%)
	Above 25	210 (72.2%)
Chronic diseases	Yes	101 (34.7%)
	No	190 (65.3%)
APAP	Yes	221 (75.9%)
NSAIDs	Yes	79 (27.1%)
Herbs	Yes	106 (36.4%)
Hot comprises	Yes	42 (14.4%)
Rx for centrally acting medication CAM	Yes	56 (19.2%)
Fulfills FM screening score	Yes	123 (42.3%)
Severe depression score	Yes	170 (58.4%)
Severe insomnia score	Yes	159 (54.6%)
PTSD mean (± SD)	Mean (± standard deviation)	30.57 (± 18.06)

### Association between analgesic classes and the participants’ factors

A Chi-square analysis revealed significant associations between analgesic groups and various categorical participant variables. Specifically, the use of acetaminophen was associated with education level and the diagnosis of chronic diseases (p < 0.05). NSAID use was associated with age, body mass index (BMI), severity of fibromyalgia symptoms, severity of insomnia symptoms, and severity of depressive symptoms (p < 0.05). The use of hot compresses was associated with age, education level, BMI, diagnosis of chronic diseases, severity of fibromyalgia symptoms, severity of insomnia symptoms, and severity of depressive symptoms (p < 0.05). Similarly, the use of CAMs was associated with age, residence, BMI, severity of fibromyalgia symptoms, severity of insomnia symptoms, and severity of depressive symptoms (p < 0.05). Additionally, an independent t-test analysis indicated no significant difference in PTSD scores between acetaminophen users (30.71 ± 18.10) and non-users (30.12 ± 18.07), t(287) = − 0.24, p = 0.81. However, users of NSAIDs reported significantly higher PTSD scores (39.40 ± 15.93) compared to non-users (27.30 ± 17.70), t(287) = − 5.29, p < 0.001. Similarly, users of hot compresses reported significantly higher PTSD scores (36.00 ± 20.04) compared to non-users (29.62 ± 17.57), t(287) = − 2.17, p = 0.03. Users of herbal household remedies showed slightly higher PTSD scores (32.18 ± 18.01) compared to non-users (29.63 ± 18.07), although the difference was not statistically significant, t(287) = − 1.16, p = 0.25. However, participants receiving a prescription of CAMs reported significantly higher PTSD scores (39.84 ± 16.37) compared to non-users (28.34 ± 17.78), t(287) = − 4.41, p < 0.001. Please refer to Table 2 for detailed results.

**Table 2:** Participants’ demographics and details across the different analgesics used

Factor	APAP	p value	NSAIDs	p value	Hot comprises	p value	Herbals	p value	Rx of central medications	p value
Age										
Below 35 years	100 (45.2%)	0.06	25 (31.6%)	< 0.001*	14 (33.3%)	0.03*	44 (41.5%)	0.06	10 (17.9%)	< 0.001*
35 years and above	121 (54.8%)		54 (68.4%)		28 (66.7%)		62 (58.5%)		46 (82.1%)	
Marital status										
Single	50 (22.6%)	0.05	18 (22.8%)	0.3	13 (31%)	0.05	22 (20.8%)	0.5	15 (26.8%)	0.12
Married	171 (77.4%)		61 (77.2%)		29 (69%)		84 (79.2%)		41 (73.2%)	
Education										
Below high school	133 (60.2%)	0.008*	41 (51.9%)	0.23	30 (71.4%)	0.02*	63 (59.4%)	0.22	36 (64.3%)	0.11
High school and above	88 (39.8%)		38 (48.1%)		12 (28.6%)		43 (40.6%)		20 (35.7%)	
Residence										
Amman	91 (41.2%)	0.37	38 (48.1%)	0.12	20 (47.6%)	0.26	41 (38.7%)	0.45	16 (28.6%)	0.03*
Peripheries	130 (58.8%)		41 (51.9%)		22 (52.4%)		65 (61.3%)		40 (71.4%)	
Body mass index										
25 and below	61 (27.6%)	0.49	11 (13.9%)	< 0.001*	11 (26.2%)	0.48	24 (22.6%)	0.09	7 (12.5%)	0.002*
Above 25	160 (72.4%)		68 (86.1%)		31 (73.8%)		82 (77.4%)		49 (87.5%)	
Chronic diseases										
No	69 (31.2%)	0.02*	19 (24.1%)	0.01*	6 (14.3%)	0.001*	31 (29.2%)	0.08	7 (12.5%)	< 0.001*
Yes	152 (68.8%)		60 (75.9%)		36 (85.7%)		75 (70.8%)		49 (87.5%)	
Fibromyalgia										
Below threshold	124 (56.1%)	0.19	31 (64.6%)	< 0.001*	24 (57.1%)	0.53	54 (50.9%)	0.05	22 (39.3%)	0.002*
Above threshold	97 (43.9%)		48 (60.8%)		18 (42.9%)		52 (49.1%)		34 (60.7%)	
Severe insomnia										
Below threshold	97 (43.9%)	0.23	24 (30.4%)	< 0.001*	15 (35.7%)	0.11	42 (39.6%)	0.09	11(19.6%)	< 0.001*
Above threshold	124 (56.1%)		55 (69.6%)		27 (64.3%)		64 (60.4%)		45 (80.4%)	
Severe depression										
Below threshold	94 (42.5%)	0.33	20 (25.3%)	< 0.001*	12 (28.6%)	0.04*	39 (36.8%)	0.13	11 (19.6%)	< 0.001*
Above threshold	57 (57.5%)		59 (74.7%)		30 (71.4%)		67 (63.2%)		45 (80.4%)	

Chi-square analysis to examine the association between the participants’ factors and the different analgesics used. Fibromyalgia symptoms was assessed using Patient self-report survey for the assessment of fibromyalgia with a cut-off score of 13 consistent with fibromyalgia burden. Insomnia severity was measured using the Insomnia severity index with a cut-off score of 14 for severe insomnia. Depression was measured using the Patient Health Questionnaire (PHQ-9) with a cut-off score of 14 for severe depression

*APAP* acetaminophen, *NSAIDs* nonsteroidal anti0inflammatory drugs

*p < 0.05

### Multivariate analysis

Multivariate binary logistic regression models were employed to assess the association between analgesic use and various mental health outcomes. For severe depressive symptoms, the final model included age, chronic diseases, NSAIDs, and CAMs. Only NSAIDs (2.07, 95% CI [2.18–3.81], p = 0.02) and CAMs (2.74, 95% CI [1.30–5.76], p = 0.008) demonstrated a significant association, indicating that participants using NSAIDs and CAMs had increased odds of experiencing severe depressive symptoms.

Regarding fibromyalgia symptoms, the final multivariate binary logistic regression model included age, chronic diseases, and NSAIDs. NSAIDs (3.03, 95% CI [1.58–5.80], p = 0.001), chronic diseases (2.48, 95% CI [1.36–4.53], p < 0.001), and age above 35 years (2.09, 95% CI [1.17–3.73], p = 0.006) exhibited significant associations, indicating that NSAID users, participants diagnosed with chronic diseases, and participants older than 35 years had higher odds of experiencing fibromyalgia symptoms.

For severe insomnia, the final multivariate binary logistic regression model included age, chronic diseases, NSAIDs, and CAMs. Only CAMs (3.90, 95% CI [2.04–5.61], p < 0.001) demonstrated a significant association, indicating that participants receiving CAMs had higher odds of experiencing severe insomnia.

Lastly, the multivariate linear regression model for PTSD included age, chronic diseases, hot compresses, NSAIDs, and CAMs. In the final model, both CAMs (β = 8.99, p = 0.001) and NSAIDs (β = 10.39, p < 0.001) showed significant associations with PTSD, suggesting that users of NSAIDs and CAMs were more likely to have higher PTSD scores. Please refer to Table 3 for detailed results.

**Table 3:** Multivariate binary logistic regression models for each dependent variable showing the significantly associated covariates

Factor	aOR	95% CI
Severe depression		
Prescription of “CAMs”	2.74	1.30–5.76
Using OTC NSAIDs	2.07	2.12–3.81
Fibromyalgia		
Using OTC NSAIDs	2.36	1.36–4.11
Diagnosed with chronic diseases (hypertension and diabetes)	2.06	1.13–3.75
Age (above 35 years)	1.87	1.07–3.25
Severe insomnia		
Prescription of “CAMs”	3.90	2.04–5.61

Factor	β 95% CI for β	t
Post-traumatic stress disorder (multivariate linear regression)		
Prescription of “CAMs”	8.99 (3.91–14.08)	3.087
Using OTC NSAIDs	10.39 (5.86–14.91)	4.210

The first multivariate binary logistic regression model with severe depression (measured using the PHQ-9 scale using a cut-off score above 14 for severe depression) as the dependent variable and adjusted for prescription of “CAMs” and NSAIDs as the independent variables. The second multivariate binary logistic regression model with fibromyalgia (measured by the Patient self-report survey for the assessment of fibromyalgia with a cut-off score above 13 consistent with fibromyalgia symptoms) as the dependent variable and adjusted for NSAIDs, chronic diseases and age as independent variable. The third multivariate binary logistic regression model with severe insomnia as the dependent variable (measures by the ISI-A scale using a cut-off score above 14 for severe insomnia) and adjusted for prescription of CAM as the independent variable. The fourth multivariate linear regression model with PTSD as the dependent variable (measured by the Davidson trauma scale (DTS)-DSM-IV) and adjusted for prescription of CAD and the use of NSAIDs as the independent variables

*aOR* adjusted odd ratio, *CAM* centrally acting medication, *NSAIDs* non-steroidal anti-inflammatory drugs confidence intervals were set at 95% and significance at p < 0.05

## Discussion

This study aimed to examine the association between OTC analgesics and the severity of subjective fibromyalgia, depression, insomnia, and PTSD symptoms in a cohort of war-displaced female Syrian refugees.

We report a high prevalence of fibromyalgia, depression, anxiety, and insomnia in our study sample. The use of NSAIDs was associated with fibromyalgia symptoms. In addition, we report that NSAIDs and CAMs were associated with severe depression, severe insomnia symptoms, and severe PTSD symptoms. The use of APAP was not associated with any of the outcome variables.

War-displaced traumatized refugees experience chronic psychological stress that starts from the moment of displacement and continues after migration and settlement [27]. This stress accompanied by neuroendocrine alterations is a leading predisposing factor to developing many physical and mental disorders such as fibromyalgia and other related mental disturbances [28]. Furthermore, the awareness of the health care providers about fibromyalgia is limited, therefore, the majority of the subjects rely on OTC analgesics for symptomatic management. This explains the high rates of fibromyalgia in this study in comparison to normal displaced populations [7].

Fibromyalgia, a central pain syndrome is best managed by antiepileptic or antidepressants according to the ACR guidelines [29].

In the present study, the majority of the participants utilized APAP. This finding is consistent with our previous results, which indicated APAP as the most commonly used medication for pain [7, 12]. This could reflect the role of healthcare providers in directing patients towards APAP, as it is relatively safer with fewer contraindications. APAP may also be chosen due to its availability or lower price [30, 31]. The present study revealed no association between APAP and fibromyalgia or other mental health symptoms, which is consistent with our previous work where APAP was not associated with PTSD among Syrian refugee females [12].

The use of NSAIDs was associated with more severe depression, insomnia, PTSD, and fibromyalgia symptoms in our study sample. NSAIDs exert their analgesic and anti-inflammatory actions by peripherally inhibiting cyclooxygenase (COX), the enzyme responsible for prostaglandin synthesis, which makes NSAIDs superior to APAP in alleviating peripheral pain [32]. In the current study, vulnerable females suffering from fibromyalgia symptoms also reported poor mental health outcomes. Therefore, our findings can be explained by the fact that females with severe fibromyalgia and poor mental health symptoms were more likely to use NSAIDs as a more effective option compared to APAP for relieving chronic widespread pain symptoms [33, 34]. Our previous study also showed that the use of NSAIDs is associated with poor fibromyalgia and mental health outcomes [7].

The ACR guidelines recommend the use of CAMs, such as anti-seizure medications or antidepressants, in the treatment of fibromyalgia [18, 35]. In our study, the use of CAMs was associated with severe depression, insomnia, and PTSD, but not with fibromyalgia. The term "CAM" refers to a prescription of centrally acting or "neurological medication for pain," as explained by the participants. This indicates the use of antiseizure or SNRI medications dispensed from the Caritas pharmacy unit. Approximately 19% of our study sample used CAMs, and their use was not associated with severe fibromyalgia symptoms. This finding is consistent with the ACR guidelines. It represents an improvement in healthcare quality compared to previous studies where only 1% of the same study population reported receiving a prescription for CAM for fibromyalgia [7]. Fibromyalgia in refugees is closely related to depression, insomnia, and PTSD [4]. Users of CAMs may have been diagnosed with fibromyalgia and may also be experiencing mental distress [4, 9]. This could explain the association between CAM use and severe depression, insomnia, and PTSD, suggesting that CAMs may alleviate fibromyalgia symptoms but no other related symptoms.

Our work adds to the existing literature on the impact of analgesics on fibromyalgia and mental health outcomes in war-displaced female refugees. The novelty of the idea, unique sample recruitment, inclusion criteria, and use of validated scales were strengths in the study design. However, the study has some limitations. The self-completing questionnaire may lack accuracy, especially for fibromyalgia. Proper diagnosis of fibromyalgia is challenging and costly, which is particularly true in low-income countries hosting war refugees. However, the self-reported scales were previously used to screen fibromyalgia and provide guidance [14–16]. In this study Based on the 2011 modification of the ACR preliminary diagnostic criteria for fibromyalgia, individuals who reported experiencing symptoms at a similar level for at least 3 months, with a score equal to or greater than 13 points (7 or higher points on the widespread pain scale and 5 or more on the severity scale and symptoms that persist for 3 months) and the absence of a medical condition explaining the symptoms, were positively screened for fibromyalgia.

Another limitation was the low response rate of the participants, which may reflect the challenges of maintaining an updated database and the dynamic nature of refugees' social circumstances, including changes in residence and phone numbers. Furthermore, complete medical profiles and detailed information regarding the NSAIDs and CAMs, such as exact names and doses, were challenging to obtain from the refugees, as they may seek medical help from different NGOs, resulting in fragmented medical records.

## Conclusion

The use of suboptimal fibromyalgia treatments is associated with poor fibromyalgia, depression, anxiety, insomnia, and PTSD self-reported symptoms in female refugees. Therefore, efforts are required to raise awareness among patients and healthcare providers regarding the proper management of fibromyalgia among this vulnerable group.

### Supplementary Information

Below is the link to the electronic supplementary material.

Supplementary file1 (PDF 341 kb)

## Data Availability

For inquiries related to data access, please contact the corresponding author's email.
