# Supplementary material for: The crosstalk between subjective fibromyalgia, mental health symptoms and the use of over-the-counter analgesics in female Syrian refugees: a cross-sectional web-based study
Source: Rheumatol Int. 2024 Jan 29;44(4):715–23. doi: 10.1007/s00296-023-05521-0 (PMC10914905; doi:10.1007/s00296-023-05521-0)
Supplement: Supplementary file 1 — Supplementary file1 (PDF 341 kb) [file 296_2023_5521_MOESM1_ESM.pdf]

Patient Health Questionnaire (PHQ-9)

Name: ____ Date: ______________________________

Over the last 2 weeks, how often have you been bothered by any of the following problems?	Not at all	Several days	More than half the days	Nearly every day
1. Little interest or pleasure in doing things	0	1	2	3
2. Feeling down, depressed, or hopeless	0	1	2	3
3. Trouble falling or staying asleep, or sleeping too much	0	1	2	3
4. Feeling tired or having little energy	0	1	2	3
5. Poor appetite or overeating	0	1	2	3
6. Feeling bad about yourself – or that you are a failure or have let yourself or your family down	0	1	2	3
7. Trouble concentrating on things, such as reading the newspaper or watching television	0	1	2	3
8. Moving or speaking so slowly that other people could have noticed? Or the opposite – being so fidgety or restless that you have been moving around a lot more than usual	0	1	2	3
9. Thoughts that you would be better off dead or of hurting yourself in some way	0	1	2	3

For office coding: Total Score _______ = ______ + ______ + ______

Total Score ______

If you checked off any problems, how difficult have these problems made it for you to do your work, take care of things at home, or get along with other people?

☐ Not difficult at all ☐ Somewhat difficult ☐ Very difficult ☐ Extremely difficult

How to Score the PHQ-9

Major depressive disorder (MDD) is suggested if:

- Of the 9 items, 5 or more are checked as at least 'more than half the days'
- Either item 1 or 2 is checked as at least 'more than half the days'

Other depressive syndrome is suggested if:

- Of the 9 items, between 2 to 4 are checked as at least 'more than half the days'
- Either item 1 or 2 is checked as at least 'more than half the days'

PHQ-9 scores can be used to plan and monitor treatment. To score the instrument, tally the numbers of all the checked responses under each heading (not at all=0, several days=1, more than half the days=2, and nearly every day=3). Add the numbers together to total the score on the bottom of the questionnaire. Interpret the score by using the guide listed below.

**Guide for Interpreting PHQ-9 Scores**		
Score	Depression Severity	Action
0 - 4	None-minimal	Patient may not need depression treatment.
5 - 9	Mild	Use clinical judgment about treatment, based on patient's duration of symptoms and functional impairment.
10 - 14	Moderate	Use clinical judgment about treatment, based on patient's duration of symptoms and functional impairment.
15 - 19	Moderately severe	Treat using antidepressants, psychotherapy or a combination of treatment.
20 - 27	Severe	Treat using antidepressants with or without psychotherapy.

Functional Health Assessment

The instrument also includes a functional health assessment. This asks the patient how emotional difficulties or problems impact work, life at home, or relationships with other people. Patient response of 'very difficult' or 'extremely difficult' suggest that the patient's functionality is impaired. After treatment begins, functional status and number score can be measured to assess patient improvement.

Note: Depression should not be diagnosed or excluded solely on the basis of a PHQ-9 score. A PHQ-9 score ≥ 10 has a sensitivity of 88% and a specificity of 88% for major depression.[1] Since the questionnaire relies on patient self-report, the practitioner should verify all responses. A definitive diagnosis is made taking into account how well the patient understood the questionnaire, as well as other relevant information from the patient.

PHQ-9 is adapted from PRIME MD TODAY, developed by Drs Spitzer, Williams, Kroenke and colleagues, with an educational grant from Pfizer Inc.
Use of the PHQ-9 may only be made in accordance with the Terms of Use available at www.pfizer.com. Copyright © 1999 Pfizer Inc. All rights reserved.
PRIME MD TODAY is a trademark of Pfizer Inc.

Reference: Kroenke K, Spitzer RL, Williams JB. The PHQ-9: Validity of a brief depression severity measure. J Gen Intern Med. 2001;16(9):606-613.

Patient Self-report Survey for the Assessment of Fibromyalgia

① Please indicate if you have had pain or tenderness **during the past 7 days** in the areas shown below.
Check the boxes in the diagram for each area in which you have had pain or tenderness.

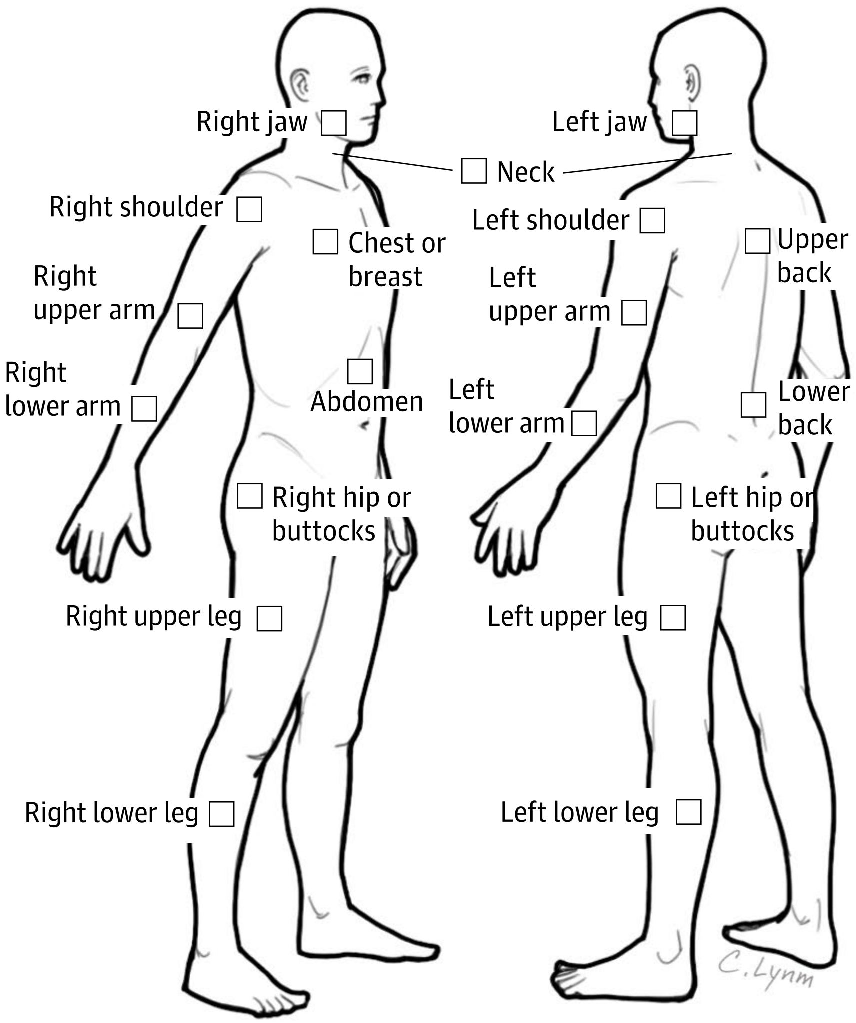

② For each symptom listed below, use the following scale to indicate the severity of the symptom **during the past 7 days**.

- **No problem**
- **Slight or mild problem:** generally mild or intermittent
- **Moderate problem:** considerable problems; often present and/or at a moderate level
- **Severe problem:** continuous, life-disturbing problems

	No problem	Slight or mild problem	Moderate problem	Severe problem
A. Fatigue	☐	☐	☐	☐
B. Trouble thinking or remembering	☐	☐	☐	☐
C. Waking up tired (unrefreshed)	☐	☐	☐	☐

③ During the **past 6 months** have you had any of the following symptoms?

A. Pain or cramps in lower abdomen	☐ No	☐ Yes
B. Depression	☐ No	☐ Yes
C. Headache	☐ No	☐ Yes

④ Have the symptoms in questions 2-3 and pain been present at a similar level for at **least 3 months**?

☐ No ☐ Yes

⑤ Do you have a disorder that would otherwise explain the pain?

☐ No ☐ Yes

© 2014 American Medical Association. All rights reserved.

Example of a Patient Self-report Survey for the Assessment of Fibromyalgia Based on Criteria in the 2011 Modification of the ACR Preliminary Diagnostic Criteria for Fibromyalgia[a]

CLINICIAN VERSION

Widespread Pain Index (1 point per check box; score range: 0-19 points)

(1) Please indicate if you have had pain or tenderness **during the past 7 days** in the areas shown below.

Check the boxes in the diagram for each area in which you have had pain or tenderness.

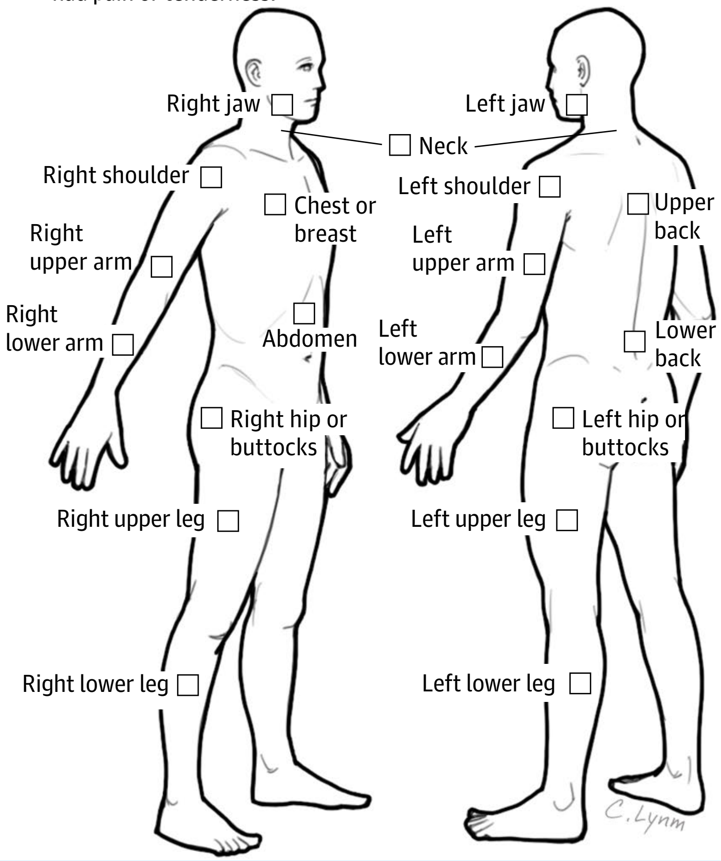

Widespread Pain Index Total (maximum, 19 points) ____

Symptom Severity (score range: 0-12 points)

(2) Using the following scale, indicate for each item your severity over the past week by checking the appropriate box.

No problem

Slight or mild problem: generally mild or intermittent

Moderate problem: considerable problems; often present and/or at a moderate level

Severe problem: continuous, life-disturbing problems

	No problem	Slight or mild problem	Moderate problem	Severe problem
A. Fatigue	☐ =0	☐ =1	☐ =2	☐ =3
B. Trouble thinking or remembering	☐ =0	☐ =1	☐ =2	☐ =3
C. Waking up tired (unrefreshed)	☐ =0	☐ =1	☐ =2	☐ =3

Subtotal (maximum, 9 points) ____

(3) During the **past 6 months** have you had any of the following symptoms?

A. Pain or cramps in lower abdomen	☐ **No** =0	☐ **Yes** =1
B. Depression	☐ **No** =0	☐ **Yes** =1
C. Headache	☐ **No** =0	☐ **Yes** =1

Subtotal (maximum, 3 points) ____

Symptom Severity Score Total (maximum, 12 points) ____

The following questions do not receive a score, but are criteria to be considered as part of the diagnostic assessment.

(4) Have the symptoms in questions 2-3 and pain been present at a similar level for at **least 3 months**?

☐ **No** ☐ **Yes**

(5) Do you have a disorder that would otherwise explain the pain?

☐ **No** ☐ **Yes**

Total (maximum, 31 points) ____

Scoring information is shown in blue. The possible score ranges from 0 to 31 points. A score equal to or greater than 13 points is consistent with a diagnosis of fibromyalgia. In addition to a cutpoint of 13 points, diagnostic criteria in the 2011 Modification of the ACR preliminary diagnostic criteria for fibromyalgia[a] specify the presence of the following 3 conditions: [1] Widespread Pain Index ≥7 and Symptom Severity ≥5 or Widespread Pain Index between 3 and 6 and Symptom Severity ≥9; [2] Presence of symptoms at a similar level for at least 3 months; [3] The patient has no other disorder to explain the pain.

[a]Reference: Wolfe F, Clauw DJ, Fitzcharles MA, et al. Fibromyalgia criteria and severity scales for clinical and epidemiological studies: a modification of the ACR preliminary diagnostic criteria for fibromyalgia. *J Rheumatol*. 2011;38(6):1113-1122.

© 2014 American Medical Association. All rights reserved.

GAD-7 Anxiety

Over the last two weeks, how often have you been bothered by the following problems?	Not at all	Several days	More than half the days	Nearly every day
1. Feeling nervous, anxious, or on edge	0	1	2	3
2. Not being able to stop or control worrying	0	1	2	3
3. Worrying too much about different things	0	1	2	3
4. Trouble relaxing	0	1	2	3
5. Being so restless that it is hard to sit still	0	1	2	3
6. Becoming easily annoyed or irritable	0	1	2	3
7. Feeling afraid, as if something awful might happen	0	1	2	3

Column totals _____ + _____ + _____ + _____ =

Total score ________

If you checked any problems, how difficult have they made it for you to do your work, take care of things at home, or get along with other people?

Not difficult at all	Somewhat difficult	Very difficult	Extremely difficult
☐	☐	☐	☐

Source: Primary Care Evaluation of Mental Disorders Patient Health Questionnaire (PRIME-MD-PHQ). The PHQ was developed by Drs. Robert L. Spitzer, Janet B.W. Williams, Kurt Kroenke, and colleagues. For research information, contact Dr. Spitzer at ris8@columbia.edu. PRIME-MD® is a trademark of Pfizer Inc. Copyright© 1999 Pfizer Inc. All rights reserved. Reproduced with permission

Scoring GAD-7 Anxiety Severity

This is calculated by assigning scores of 0, 1, 2, and 3 to the response categories, respectively, of “not at all,” “several days,” “more than half the days,” and “nearly every day.”
GAD-7 total score for the seven items ranges from 0 to 21.

0–4: minimal anxiety

5–9: mild anxiety

10–14: moderate anxiety

15–21: severe anxiety

Insomnia Severity Index

The Insomnia Severity Index has seven questions. The seven answers are added up to get a total score. When you have your total score, look at the 'Guidelines for Scoring/Interpretation' below to see where your sleep difficulty fits.

For each question, please CIRCLE the number that best describes your answer.

Please rate the CURRENT (i.e. LAST 2 WEEKS) SEVERITY of your insomnia problem(s).

Insomnia Problem	None	Mild	Moderate	Severe	Very Severe
1. Difficulty falling asleep	0	1	2	3	4
2. Difficulty staying asleep	0	1	2	3	4
3. Problems waking up too early	0	1	2	3	4

4. How SATISFIED/DISSATISFIED are you with your CURRENT sleep pattern?

Very Satisfied	Satisfied	Moderately Satisfied	Dissatisfied	Very Dissatisfied
0	1	2	3	4

5. How NOTICEABLE to others do you think your sleep problem is in terms of impairing the quality of your life?

Not at all Noticeable	A Little	Somewhat	Much	Very Much Noticeable
0	1	2	3	4

6. How WORRIED/DISTRESSED are you about your current sleep problem?

Not at all Worried	A Little	Somewhat	Much	Very Much Worried
0	1	2	3	4

7. To what extent do you consider your sleep problem to INTERFERE with your daily functioning (e.g. daytime fatigue, mood, ability to function at work/daily chores, concentration, memory, mood, etc.) CURRENTLY?

Not at all Interfering	A Little	Somewhat	Much	Very Much Interfering
0	1	2	3	4

Guidelines for Scoring/Interpretation:

Add the scores for all seven items (questions 1 + 2 + 3 + 4 + 5 +6 + 7) = _______ your total score

Total score categories:
0–7 = No clinically significant insomnia
8–14 = Subthreshold insomnia
15–21 = Clinical insomnia (moderate severity)
22–28 = Clinical insomnia (severe)

Used via courtesy of www.myhealth.va.gov with permission from Charles M. Morin, Ph.D., Université Laval
